# Genetic and clinical determinants of MACE and haemorrhage in antiplatelet therapy: insights from pharmacogenomic analysis

**DOI:** 10.3389/fcvm.2025.1572389

**Published:** 2025-05-12

**Authors:** Yubo Wang, Shuangli Yuan, Muyun Li, Wenling Feng, Jing Li, Wenwen Chen, Aliye Berdi, Yulian Kou, Yuan Yuan, Jun Zhao

**Affiliations:** ^1^ Department of Pharmacy, The First Affiliated Hospital of Xinjiang Medical University, Urumqi, Xinjiang Uygur Autonomous Region, China; ^2^ Xinjiang Key Laboratory of Clinical Drug Research, Urumqi, Xinjiang Uygur Autonomous Region, China; ^3^ Department of Pharmacy, The Sixth Affiliated Hospital of Xinjiang Medical University, Urumqi, Xinjiang Uygur Autonomous Region, China; ^4^ Pharmacy School, Xinjiang Medical University, Urumqi, Xinjiang Uygur Autonomous Region, China

**Keywords:** antiplatelet therapy, clopidogrel, pharmacogenomics, multigene testing, personalized medicine, cardiovascular events

## Abstract

**Background:**

Variability in responses to clopidogrel and aspirin therapy for coronary artery disease has driven interest in pharmacogenomics. This study investigates the role of genetic variants in *CYP2C19*, *ABCB1*, and *PON1* in predicting adverse cardiovascular events and guiding personalised antiplatelet therapy.

**Methods:**

A retrospective cohort study designed to compare the effectiveness and safety of the risk levels from *CYP2C19* (*2, *3, *17), *ABCB1 C3435T*, and *PON1 Q192R* polymorphisms. The primary outcome was the incidence of haemorrhage and major adverse cardiovascular events (MACE). Kaplan Merir curves and Cox regression with IPTW adjustments were used for analysis.

**Results:**

The results of this study indicate that patients in Group A, who received treatment consistent with multigene testing (*CYP2C19*, *ABCB1*, and *PON1*), experienced significantly lower major adverse cardiovascular events (MACE) compared to Group B. Multigene testing proved to be more accurate in predicting clopidogrel effectiveness and reducing adverse events without an increased risk of haemorrhage (HR 0.671, 95% CI: 0.526–0.855, *P* = 0.001). Patients in Group A showed no significant difference in haemorrhage risk compared to Group B, with an HR of 0.831 (95% CI: 0.598–1.155, *P* = 0.271) after adjustment.

**Conclusion:**

Multigene-guided antiplatelet therapy is more effective in reducing adverse cardiovascular events. Further prospective studies are needed to validate these findings, incorporating genetic, environmental, and lifestyle factors for a comprehensive personalised medicine approach.

## Introduction

As medical research develops in depth, the significance of antiplatelet drugs in the treatment of coronary artery disease (CAD) has increasingly come into focus. Among these, clopidogrel, in combination with aspirin, constitutes the cornerstone of CAD therapy, widely and routinely used in clinical practice and recommended by guidelines (1). Although clopidogrel shows remarkable clinical efficacy, there is significant variability in patient responses to the same dosage, and this interindividual heterogeneity has attracted widespread attention in the medical community. Studies have found that approximately 4%–30% of patients exhibit poor antiplatelet effects after receiving standard doses of clopidogrel, leading to the so-called “clopidogrel resistance” phenomenon (2).

The variability in clopidogrel's therapeutic effects results from a combination of genetic and non-genetic factors (3). Genome-wide association studies have revealed that over 83% of the individual variability in clopidogrel response may be regulated by genetic effects related to the enzymes involved in its absorption and metabolism pathways (4). As a prodrug, clopidogrel's absorption, metabolism, and action involve the expression of numerous crucial genes. The *ABCB1* gene encodes P-glycoprotein, which is responsible for the transport and absorption of the drug (5), while the CYP2C19 and *PON1* genes directly affect the metabolic activation of clopidogrel (6). The polymorphism of the *CYP2C19* gene is particularly noteworthy since the enzyme it encodes plays an indispensable role in the metabolism of clopidogrel (7), and its allelic variations, such as CYP2C19*2, *3, and *17, directly affect the efficacy and metabolic rate of clopidogrel (8, 9).

In studies on the relationship between major adverse cardiovascular events (MACE) and clopidogrel treatment, the role of *CYP2C19* gene polymorphism cannot be underestimated (10, 11). The U.S. Food and Drug Administration (FDA) pointed out as early as 2010 that the loss-of-function alleles of *CYP2C19* are associated with poor response to clopidogrel therapy and required a warning in the drug labelling. Drug labels in China similarly indicate potential issues for slow metabolizers of *CYP2C19* when using clopidogrel (12). Recent research further supports the importance of genotype-guided therapy in improving the efficacy and safety of clopidogrel treatment, especially in patients with ST-segment elevation myocardial infarction (STEMI), where genotype guidance can significantly reduce the risk of recurrent myocardial infarction, cardiovascular death, and major bleeding (13). Moreover, the latest meta-analyses also show that genotype-guided antiplatelet therapy can significantly improve treatment outcomes compared to standard therapy, particularly in the Chinese population or patients with acute coronary syndrome (ACS) (14, 15).

However, the polymorphisms of the *CYP2C19* gene cannot fully account for the interindividual variability, suggesting that the individual differences in the therapeutic efficacy of clopidogrel are the complex result of multigenic interactions (4). Some studies have indicated that in addition to *CYP2C19*, the *PON1* and *ABCB1* genes also play a crucial role in the therapeutic effects of clopidogrel (16, 17). Mutations at the *C3435T* locus of the *ABCB1* gene can enhance the efflux action of P-glycoprotein, thereby reducing the effective plasma concentration of clopidogrel (18). Additionally, research has found that this mutation is associated with the *in vivo* concentration of both clopidogrel and its active metabolites (19).

Recent advances in pharmacogenomic research have underscored the need to integrate polygenic risk models in predicting interindividual variability in clopidogrel response. Accumulating evidence suggests that polymorphisms in genes regulating clopidogrel metabolism, absorption, and bioactivation significantly impact its therapeutic efficacy. Notably, the CYP2C19*2 loss-of-function allele *(rs4244285)* and the *ABCB1 C3435T* variant (rs1045642) have been robustly associated with impaired antiplatelet effects, as demonstrated in meta-analysis (20)*.* Furthermore, the CYP2C19*3 allele (rs4986893) exhibits analogous functional consequences, reducing enzymatic activity and predisposing carriers to diminished clopidogrel responsiveness (21). Conversely, the CYP2C19*17 gain-of-function variant (rs12248560) is associated with increased metabolic activation of the prodrug, resulting in enhanced platelet inhibition, as indicated by a meta-analysis (22). Beyond cytochrome P450 polymorphisms, transporter gene variants also contribute to pharmacokinetic variability. The *ABCB1 C3435T* polymorphism has been implicated in reduced intestinal P-glycoprotein efflux activity, thereby altering clopidogrel absorption dynamics and contributing to exposure variability (23). Additionally, functional studies of the paraoxonase-1 (*PON1*) *Q192R* variant (rs662) suggest its role in modulating the hydrolysis of clopidogrel's active metabolite, with the *192R* allele linked to accelerated degradation and subsequent attenuation of therapeutic efficacy (24). Recent systematic reviews have synthesized these multilocus interactions, underscoring the clinical relevance of combinatorial genetic models for optimizing clopidogrel dosing strategies.

Integrating the results from single-gene and multi-gene studies, we recognize that there is an upward trend in the risk of cardiovascular events when the *ABCB1 C3435T* and CYP2C19*2 alleles coexist (25). These findings underscore the potential value of multigene testing to predict a patient's response to clopidogrel. These findings underscore the potential value of multigene testing to predict a patient's response to clopidogrel. Thereby, we hypothesize that specific genetic variants, alongside clinical characteristics, significantly influence the risk of MACE and hemorrhage in patients undergoing antiplatelet therapy. Given this context, this study aims to investigate the genetic and clinical determinants that influence the risk of MACE and haemorrhage in patients on antiplatelet therapy, with a focus on genes involved in clopidogrel metabolisms, including *CYP2C19*, *PON1*, and *ABCB1*.

## Materials and methods

### Study design and subjects

This cohort study included patients undergoing antiplatelet therapy with coronary artery disease (CAD) who were hospitalized at the First Affiliated Hospital of Xinjiang Medical University from January 2016–December 2020, with detailed data collection on genetic profiles and clinical characteristics. The study protocol was approved by the institutional ethical review board (Approval No. K202106-24).

### Inclusion and exclusion criteria

Inclusion criteria were patients aged 18 and above receiving clopidogrel therapy, including conformed to the diagnostic criteria for coronary artery disease (1); underwent genetic testing for *CYP2C19* (26), *ABCB1*, and *PON1*; were scheduled to receive antiplatelet therapy with clopidogrel or ticagrelor in combination with aspirin. Exclusion criteria included patients with incomplete genetic data or those on alternative antiplatelet regimens: those lacking basic information, genetic testing, or other relevant test data; those with a history of severe liver or kidney dysfunction or malignant tumours; those also using other anticoagulant medications such as warfarin or rivaroxaban; those whose antiplatelet therapy lasted less than six months. Those unable to complete follow-up.

### Data collection

Data from the participating patients were collected through the medical record management system, including but not limited to gender, age, body mass index (BMI), hypertension, diabetes, smoking history, disease classification, surgical history, and diagnosis and treatment information. In addition, related biochemical indicators were collected, such as routine blood tests, liver and kidney function, lipid levels, and imaging data, such as left ventricular ejection fraction. Genomic DNA was extracted from blood samples, including the *2, *3, *17 alleles of *CYP2C19*, *ABCB1 C3435T*, and *PON1 Q192R*.

In this study, due to the complexity of real-world data and the lack of standardization, we first cleansed the dataset to ensure the accuracy and validity of the analysis. In this study, we normalized the data before analysis to ensure consistency. Data with the same meaning but different expressions were standardized into a unified format through data transformation. For variables with multiple types, we combined those with similar properties or reorganized features with a large number of categories but low frequencies into a single category to reduce the number of features, a process known as data reduction. Following this, we integrated and stored data from various tables into a standardized data table, a process known as data integration. Finally, continuous variables were converted into categorical variables with a more balanced distribution, and textual data were transformed into numerical formats for analysis. Since the data for the study came from hospital electronic medical records accessed via telephone, there was very little missing data. If important data such as medication information, genetic tests, or outcome events were missing from the electronic records, the case would be excluded from the study.

### Outcome measures and follow-up

The primary outcome measure of this study was the incidence rate of major cardiovascular adverse events (MACE) and bleeding events following treatment. Information on the patients' detailed use of antiplatelet drugs and any occurrences and timing of adverse cardiovascular events was collected through rehospitalisation records and telephone follow-ups. In this study, MACE includes cardiac death, non-fatal myocardial infarction, heart failure, revascularisation, ischemic stroke, and cardiac rehospitalisation, among others. Bleeding events are presented in Table 1 (27).

**Table 1:** Definition of bleeding classification.

Definition	Manifestation (Meets one of the following)	Haemoglobin drop Value	Transfusion of whole blood or packed red cells
Life-Threatening Bleeding	Fatal bleeding, intracranial haemorrhage, or cardiac tamponade due to haemorrhage, bleeding leading to shock or hypotension requiring vasopressor therapy or surgery	>50 g/L	≥4 U
Other Serious Bleeding	Disabling (e.g., permanent loss of vision)	30–50 g/L	2–3 U
Moderate Bleeding	Requires medical intervention for haemostasis (e.g., epistaxis requiring a device for haemostasis)		
Minor Bleeding	Other (gum bleeding, bruising, bleeding at the injection site) not requiring medical intervention		

### Group strategy

Genotyping of the *CYP2C19* gene includes wild type *1/*1, heterozygous mutants *1/*2, *1/*3, *1/*17, *2/*17, *3/*17, and homozygous mutants *2/*2, *3/*3, *2/*3, *17/*17; *ABCB1 C3435T* genotypes are categorized as wild type (*CC*), heterozygous (*CT*), and homozygous mutant (*TT*). *PON1 Q192R* genotypes are classified as wild type (*GG*), heterozygous (*GA*), and homozygous mutant (*AA*). Based on the rate of drug metabolism, *CYP2C19* genotypes are classified into four metabolizer phenotypes: ultra-rapid metabolizer (*UM*) *17/*17, *1/*17; extensive metabolizer (*EM*) *1/*1; intermediate metabolizer (IM) *1/*2, *1/*3, *2/*17, *3/*17; and poor metabolizer (*PM*) *2/*2, *2/*3, *3/*3 (9). The combined effects of CYP2C19*2, *3, *17, ABCB1 C3435T, and *PON1 Q192R* genes were categorized as 6 levels in this study: Level -1 (bleeding risk), Level 0 (standard), Level 1 (low risk of clopidogrel resistance), Level 2 (moderate risk of clopidogrel resistance), Level 3 (high risk of clopidogrel resistance), and Level 4 (very high risk of clopidogrel resistance), as detailed in Table 2.

**Table 2:** The risk level of the gene combination.

Level	CYP2C19	PON1 Q192R	ABCB1 C3435T
Level -1 (bleeding risk)	UM (*1/*17 or *17/*17)	GG	CC/CT/TT
Level 0 (normal)	EM (*1/*1)	GG	CC/CT
	UM (*1/*17 or *17/*17)	GA/AA	CC/CT/TT
Level 1 (low risk of clopidogrel resistance)	EM (*1/*1)	GG	TT
	EM (*1/*1)	GA	CC/CT
	IM (*1/*2 or *1/*3 or *2/*17 or *3/*17)	GG	CC/CT/TT
Level 2 (moderate risk of clopidogrel resistance)	EM (*1/*1)	GA	TT
	IM (*1/*2 or *1/*3 or *2/*17 or *3/*17)	GA	CC/CT/TT
Level 3 (high risk of clopidogrel resistance)	EM (*1/*1)	AA	CC/CT/TT
	IM (*1/*2 or *1/*3 or *2/*17 or *3/*17)	AA	CC/CT/TT
	PM (*2/*3)	GG/GA	CC/CT/TT
	PM(*3/*3)	GG/GA/AA	CC/CT/TT
Level 4 (very high risk of clopidogrel resistance)	PM(*2/*3 or *2/*2)	AA	CC/CT/TT

Ultra-rapid metabolizer (UM) *17/*17, *1/*17; Extensive metabolizer (EM) *1/*1; Intermediate metabolizer (IM) *1/*2, *1/*3, *2/*17, *3/*17; and Poor metabolizer (PM).

According to the guidelines for coronary heart disease and the latest expert consensus on dual antiplatelet therapy (1, 28), in conjunction with the clinical guidelines published by PharmGKB and CPIC (9), and considering the actual conditions of our institution, we have developed the following antiplatelet therapy plans. For patients at Level 1, a combined treatment plan of clopidogrel (75 mg/day) and aspirin (100 mg/day) is recommended. If the patient experiences bleeding events or is at risk of bleeding, it is advised to reduce the clopidogrel dose to 50 mg/day. At Levels 0 and 1, patients maintain a combination of clopidogrel (75 mg/day) and aspirin (100 mg/day). For patients at Level 2, ticagrelor (90 mg, twice a day) is recommended, or increasing the clopidogrel dose to 150 mg/day, combined with aspirin (100 mg/day). For patients at Levels 3 and 4, it is recommended to use ticagrelor (90 mg, twice daily) in place of clopidogrel, in combination with aspirin (100 mg/day).

Based on whether the patient's antiplatelet treatment plan aligns with the recommendations derived from the genetic testing results, patients are divided into two groups: Group A, where the treatment plan is consistent with the genetic testing recommendations; and Group B, where the treatment plan is not consistent with the genetic testing recommendations. This study aims to compare the incidence of major adverse cardiovascular events (MACE) in patients with coronary heart disease between the two treatment plans and to assess the clinical value of antiplatelet therapy guided by the results of multigene testing.

### Statistical methods

Descriptive statistics will be reported for baseline data. For continuous data, normality tests will be performed first; if each group satisfies normality and the variances between groups are equal, the *t*-test will be used for intergroup comparison; otherwise, the non-parametric Wilcoxon rank-sum test will be considered. The chi-square test was used for unordered outcomes for categorical data, and the non-parametric Wilcoxon rank-sum test was used for ordered data.

Multifactorial Cox regression models were utilized to assess the impact of genetic and clinical factors on MACE and haemorrhage outcomes, both before and after IPTW adjustment. Confounding factors were controlled to ensure robust analysis. This study adopted the propensity score to estimate the inverse probability of treatment weight (IPTW), which was achieved through the following steps: selecting covariates based on multivariate Cox regression and clinical expert opinion to identify related confounding factors. Based on past literature studies and expert opinions, 15 variables are included as confounding factors: gender, age, ethnicity, BMI, smoking status, whether diabetes was diagnosed and disease type, whether surgical procedure was given, monocyte percentage, platelet count, mean platelet volume, uric acid, low-density lipoprotein, left ventricular ejection fraction, and bleeding risk. The standardized mean difference of each selected covariate was reported. All statistical analyses will be performed using R software.

## Results

**Figure 1:**
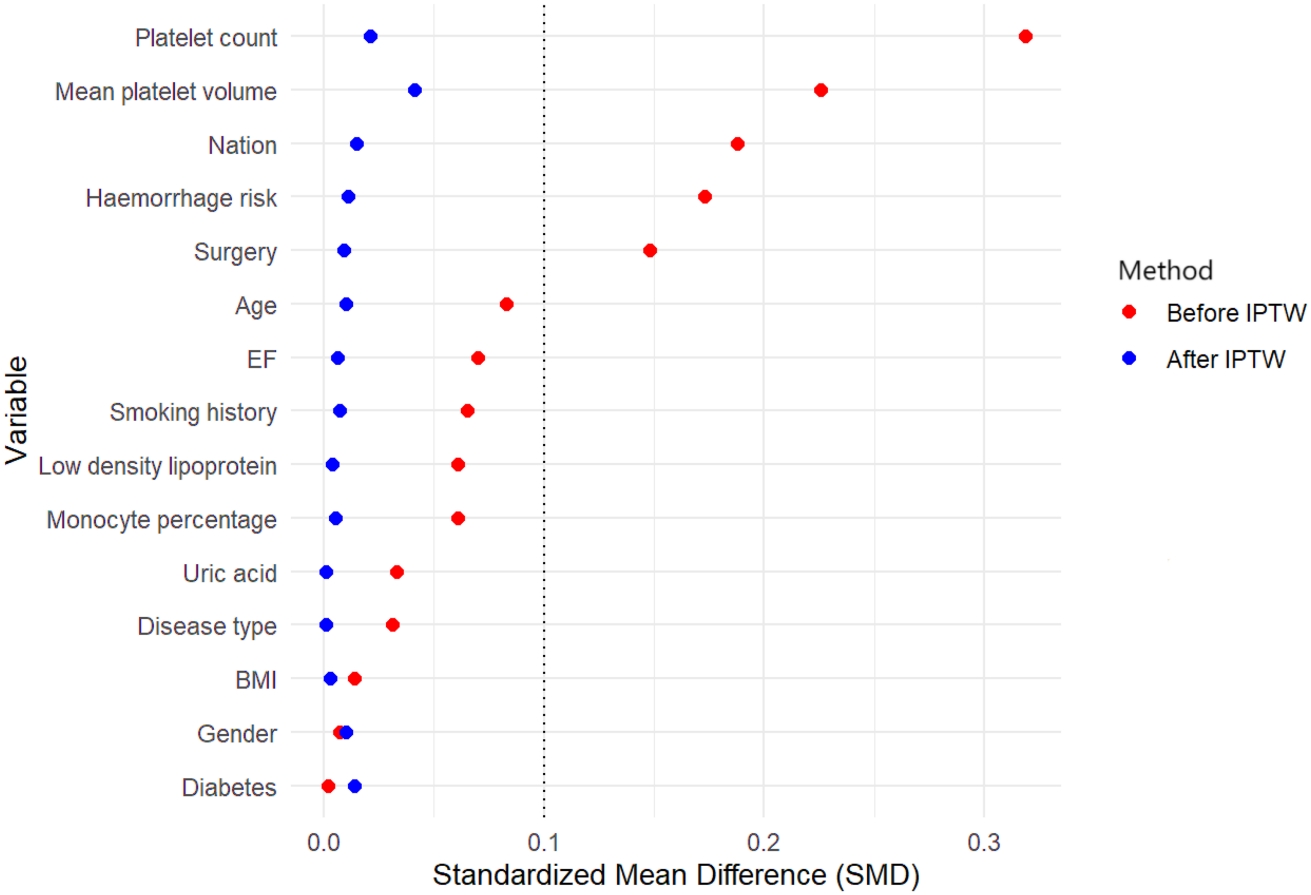
Standardized mean difference for the covariates before and after weighting. BMI, body mass index; EF, left ventricular ejection fraction; IPTW, Inverse probability of treatment weight.

The cohort consisted of 601 patients, and the distribution of genetic polymorphisms and clinical characteristics is detailed in Table 3. Of 601 patients, 85.71% of Level -1 patients, 68.87% of Level 0 patients, 79.43% of Level 1 patients, 24.22% of Level 2 patients, 31.69% of Level 3 patients, and 22.22% of Level 4 patients were classified into Group A (53.74%); 14.29% of Level -1 patients, 31.13% of Level 0 patients, 20.57% of Level 1 patients, 75.78% of Level 2 patients, 68.31% of Level 3 patients, and 77.78% of Level 4 patients were classified into Group B (46.26%). The standardized mean difference before and after IPTW were less than 0.1, indicating a well-balance of selected variables (Figure 1).

**Table 3:** The details of variable before/after adjustment by IPTW [example(%)].

Variable	A group (*n* = 323)	B group (*n* = 278)	Before IPTW			After IPTW		
			P(MACE)	P(BE)	SMD	P(MACE)	P(BE)	SMD
Gender			0.391	0.229	0.007	0.158	0.052	0.010
Female	66 (20.43)	56 (20.14)						
Male	257 (79.57)	222 (79.86)						
Age (years)			0.004	0.411	0.083	<0.001	0.249	0.011
<55	98 (30.34)	91 (32.73)						
≥55 and <65	111 (34.37)	98 (35.25)						
≥65 and <75	78 (24.15)	58 (20.86)						
≥75	36 (11.15)	31 (11.15)						
Ethnics			0.003	0.006	0.188	<0.001	<0.001	0.015
Han	217 (67.18)	168 (60.43)						
Uygur	70 (21.67)	83 (29.86)						
Other	36 (11.15)	27 (9.71)						
BMI (kg/m^2^)			0.184	0.447	0.014	0.149	0.134	0.003
<24	88 (27.24)	74 (26.62)						
≥24	235 (72.76)	204 (73.38)						
Smoking history			0.124	0.150	0.065	0.020	0.114	0.007
No	151 (46.75)	139 (50.00)						
Yes	172 (53.25)	139 (50.00)						
Diabetes			0.521	0.059	0.002	0.594	0.003	0.014
No	196 (60.68)	169 (60.79)						
Yes	127 (39.32)	109 (39.21)						
Disease type			0.049	0.492	0.031	0.003	0.233	<0.001
Chronic coronary syndrome	114 (35.29)	94 (33.81)						
Acute coronary syndrome	209 (64.71)	184 (66.19)						
Surgical intervention			0.194	0.067	0.148	0.018	0.001	0.009
No	71 (21.98)	76 (27.34)						
PCI	247 (76.47)	195 (70.14)						
CABG	5 (1.55)	7 (2.52)						
Monocyte percentage (%)			0.308	0.424	0.061	0.111	0.101	0.005
<10.00	283 (87.62)	249 (89.57)						
≥10.00	40 (12.38)	29 (10.43)						
Platelet count(10^9^/L)			0.277	0.866	0.319	0.023	1	0.021
<300.00	298 (92.26)	227 (81.65)						
≥300.00	25 (7.74)	51 (18.35)						
Mean platelet volume(fL)			0.431	0.515	0.226	0.032	0.638	0.041
<12.50	305 (94.43)	274 (98.56)						
≥12.50	18 (5.57)	4 (1.44)						
Uric acid (*μ*mol/L)			0.247	0.368	0.033	0.078	0.291	0.001
<428.00	286 (88.54)	249 (89.57)						
≥428.00	37 (11.46)	29 (10.43)						
Low density lipoprotein (mmol/L)			0.313	0.575	0.061	0.358	0.324	0.004
<1.80	121 (37.46)	96 (34.53)						
≥1.80	202 (62.54)	182 (65.47)						
Left ventricular ejection fraction (%)			0.518	0.474	0.173	0.117	0.265	0.006
Normal (≥50.00)	291 (90.09)	256 (92.09)						
Low (<50.00)	32 (9.91)	22 (7.91)						
Elevated risk of haemorrhage^a^			0.739	0.894	0.070	0.292	1	0.019
No	300 (92.88)	244 (87.77)						
Yes	23 (7.12)	34 (12.23)						

BE, bleeding events.

^a^ Elevated risk of bleeding is a composite variable defined as one or more: a history of peptic ulcer or reflux oesophagus or gastritis; history of previous haemorrhage; high blood creatinine (≥115 *μ*mol/L).

**Figure 2:**
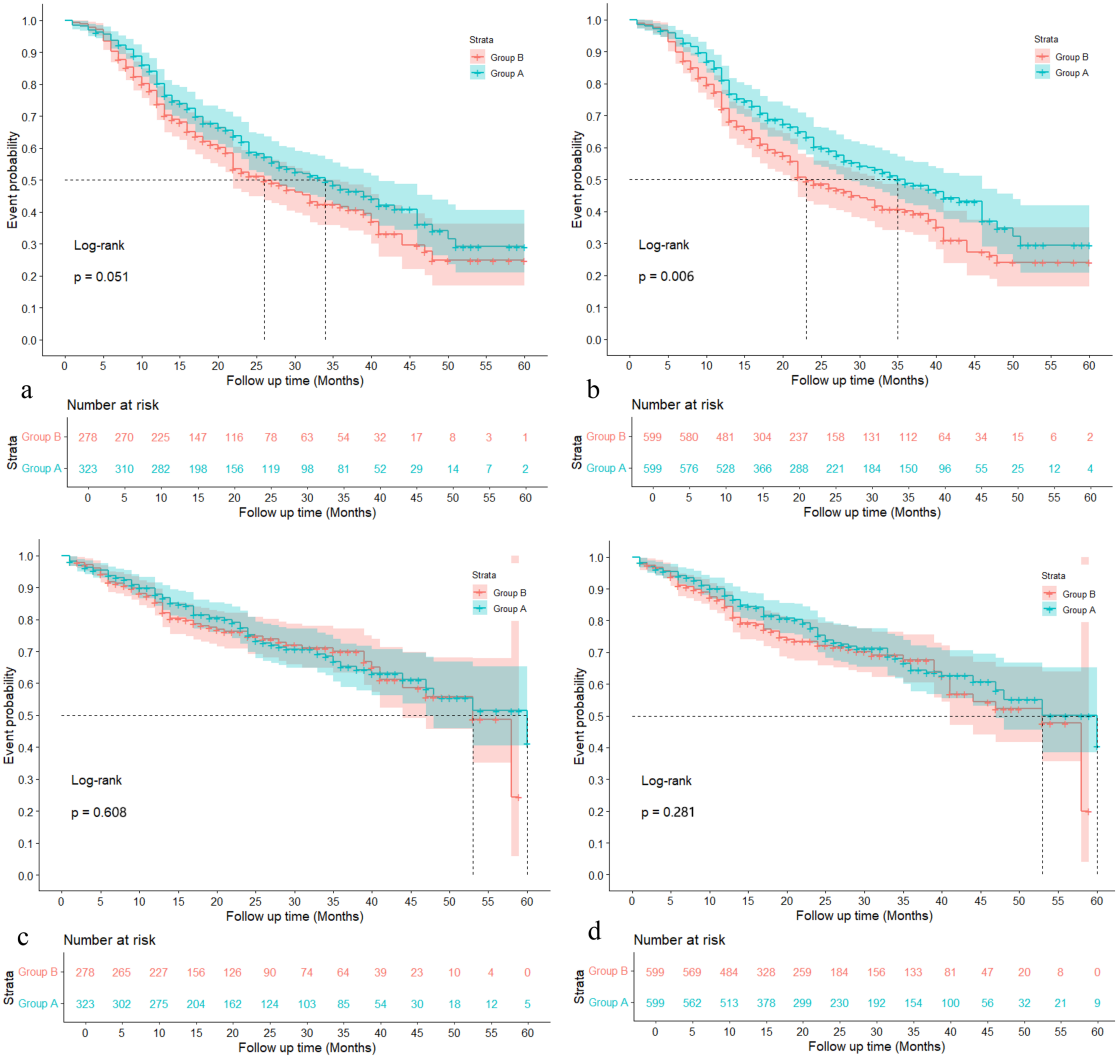
Probabilities of MACE and haemorrhagic event with the 60 months follow-up. **(a)**: MACE event probabilities during 60 months follow-up before IPTW; **(b)** MACE event probabilities during 60 months follow-up after IPTW; **(c)**: Haemorrhagic event probabilities during 60 months follow-up before IPTW; **(d)**: Haemorrhagic event probabilities during 60 months follow-up after IPTW.

Figures 2a,b demonstrate the MACE (Major Adverse Cardiovascular Event) event probabilities over a 60-month follow-up period before and after IPTW (Inverse Probability of Treatment Weighting), respectively. In Figure 2a, before applying IPTW, there is no significant difference between Group A and Group B (Log-rank *p* = 0.051), with Group A showing slightly lower event probabilities than Group B throughout the follow-up period. The number at risk decreases steadily for both groups over time, with Group A starting with 323 individuals and Group B starting with 278 individuals. In contrast, Figure 2b illustrates the MACE event probabilities after IPTW adjustment, showing a significant difference between the two groups (Log-rank *p* = 0.006). Group A again exhibits lower event probabilities than Group B, but the divergence between the curves is more pronounced post-IPTW.

Figures 2c,d illustrate the haemorrhagic event probabilities over a 60-month follow-up period before and after IPTW (Inverse Probability of Treatment Weighting), respectively. In Figure 2c, prior to IPTW adjustment, there is no significant difference between Group A and Group B regarding haemorrhagic event probabilities, with a Log-rank *p*-value of 0.608. Both groups display similar trends in event probabilities, and the number at risk decreases steadily over time, with Group A starting with 323 individuals and Group B with 278 individuals. Following the IPTW adjustment in Figure 2d, the comparison remains non-significant, although the Log-rank *p*-value improves slightly to 0.281.

**Figure 3:**
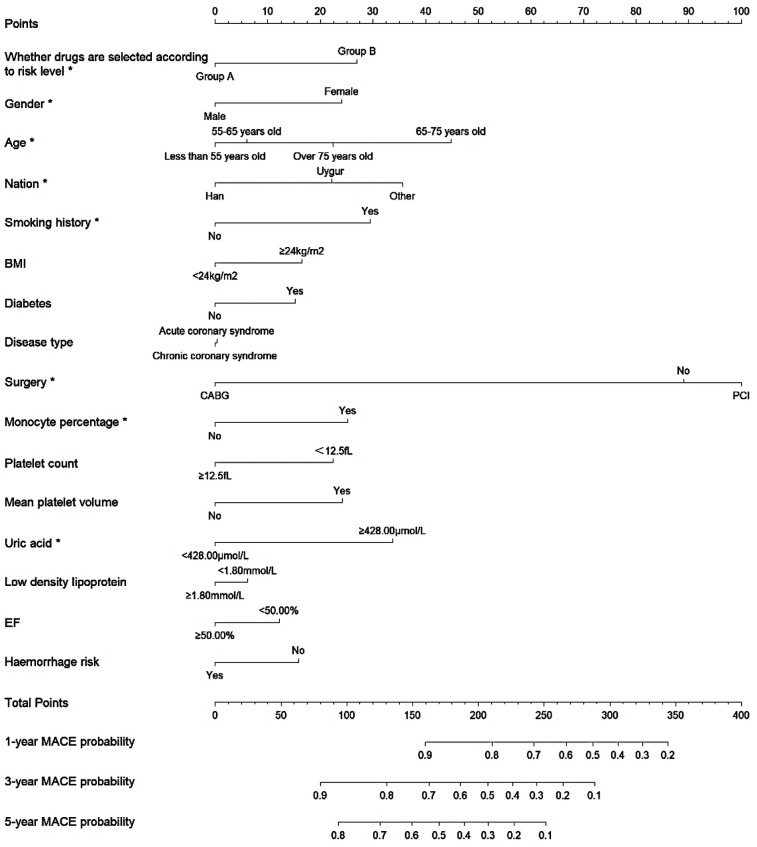
Nomogram for predicting 1-year, 3-year, and 5-year MACE probability in patients undergoing antiplatelet therapy before IPTW.

**Figure 4:**
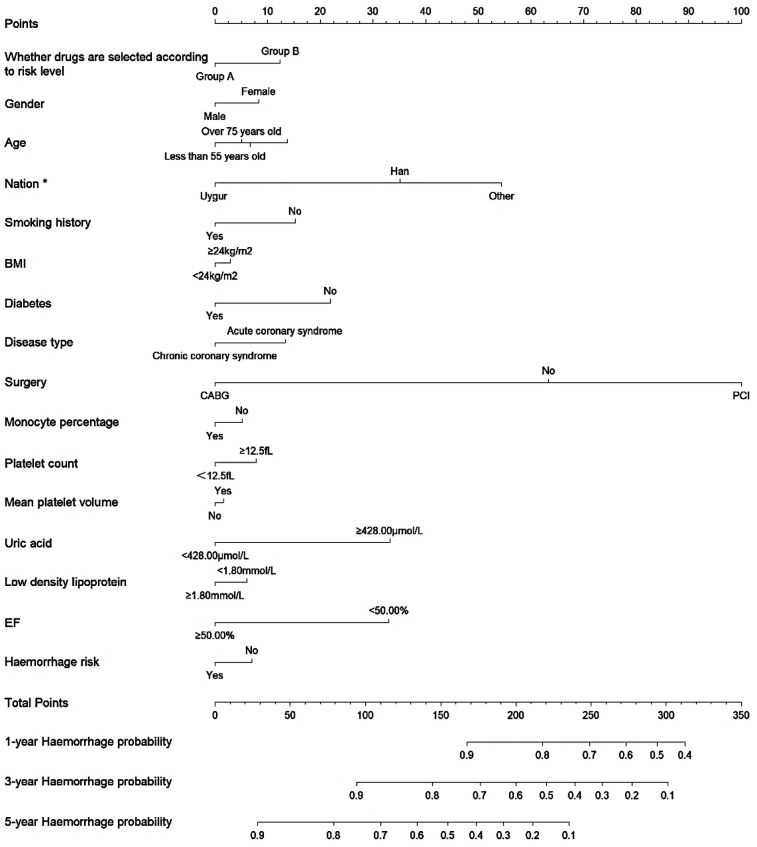
Nomogram for predicting 1-year, 3-year, and 5-year haemorrhage probability in patients undergoing antiplatelet therapy after IPTW.

The results of the multifactorial Cox regression analysis presented in Figures 3,4 reveal several significant predictors of MACE both before and after adjustment using IPTW. Patients in Group A demonstrated a significantly lower risk of MACE compared to Group B, with a hazard ratio (HR) of 0.691 (95% CI: 0.542–0.879, *P* = 0.002) before IPTW adjustment, which remained significant after IPTW adjustment (HR 0.671, 95% CI: 0.526–0.855, *P* = 0.001). Age was a notable predictor, with patients aged 65–75 years having a significantly higher risk of MACE compared to those under 55 years, both before (HR 1.860, 95% CI: 1.338–2.585, *P* < 0.001) and after IPTW adjustment (HR 1.940, 95% CI: 1.356–2.775, *P* < 0.001). Ethnicity also played a role, with Uygur and other non-Han ethnicities showing an increased risk of MACE. Uygur patients had an HR of 1.364 (95% CI: 1.027–1.812, *P* = 0.031) before IPTW and 1.388 (95% CI: 1.036–1.858, *P* = 0.027) after IPTW. Smoking was another significant factor, associated with a higher risk of MACE before (HR 1.504, 95% CI: 1.117–2.026, *P* = 0.007) and after adjustment (HR 1.546, 95% CI: 1.127–2.121, *P* = 0.006). Additionally, elevated uric acid levels (≥428.00 μmol/L) were consistently associated with an increased risk of MACE both before (HR 1.614, 95% CI: 1.121–2.324, *P* = 0.009) and after IPTW adjustment (HR 1.646, 95% CI: 1.148–2.360, *P* = 0.006). On the contrary, undergoing coronary artery bypass grafting (CABG) was associated with a lower risk of MACE, with a significant reduction in hazard before (HR 0.309, 95% CI: 0.096–0.992, *P* = 0.048) and after adjustment (HR 0.266, 95% CI: 0.087–0.814, *P* = 0.020). (Details of the results are shown in Supplementary Table S1).

The multifactorial Cox regression analysis results for haemorrhage, as presented in Figure 4, indicate that the adjusted hazard ratios (HRs) after IPTW did not substantially differ from the unadjusted analysis. Notably, patients in Group A showed no significant difference in haemorrhage risk compared to Group B, with an HR of 0.851 (95% CI: 0.615–1.177, *P* = 0.330) before IPTW adjustment and 0.831 (95% CI: 0.598–1.155, *P* = 0.271) after adjustment. Uygur ethnicity was associated with a significantly lower risk of haemorrhage both before (HR 0.604, 95% CI: 0.386–0.945, *P* = 0.027) and after IPTW adjustment (HR 0.592, 95% CI: 0.369–0.951, *P* = 0.030). On the other hand, the type of surgical intervention also showed significance, where undergoing percutaneous coronary intervention (PCI) was associated with a higher risk of haemorrhage before (HR 1.633, 95% CI: 1.087–2.452, *P* = 0.018) and after IPTW adjustment (HR 1.723, 95% CI: 1.147–2.588, *P* = 0.008). Other variables, such as age, gender, BMI, smoking status, and diabetes, did not show significant associations with haemorrhage risk after adjustment, although some trends were noted. (Details of the results of Cox regression before and after IPTW are shown in Supplementary Table S2).

## Discussion

The results of this study were integrated with existing literature to delve deeper into the predictive value of combined testing of *CYP2C19*, *ABCB1*, and *PON1* genes in determining differential responses to clopidogrel treatment. Our findings revealed that compared to the baseline (patients at level 0), the risk of adverse cardiovascular events significantly increased for patients at levels 2, 3, and 4. This reinforces the potential application of a multigene model in predicting the efficacy of clopidogrel. Specifically, we observed that antiplatelet treatment plans consistent with multigene testing results corresponded with a lower risk of adverse cardiovascular events. Building on our previous research on single-gene testing, the current results further highlight the significant value of comprehensive multigene testing in guiding personalized antiplatelet therapy, particularly in reducing adverse cardiovascular events.

Our findings align with several existing studies. A meta-analysis involving 11,740 patients (14) demonstrated that genotype-guided therapy significantly reduces the risk of all efficacy outcomes compared to standard treatment, without increasing bleeding event risks. Similarly, research by Shi et al. (29) found that individualized treatment based on the *CYP2C19* genotype reduced major adverse cardiac and cerebrovascular events in Chinese ACS patients. Another multicenter prospective study (30) that randomly assigned patients to standard treatment or pharmacogenetic testing groups revealed that patients receiving genotype-guided treatment had a significantly lower incidence of ischemic events, with no significant difference in bleeding events.

The present study revealed that the allele frequencies (MAF) of CYP2C19 *2, *3, and *17 were 27.49%, 4.84%, and 4.31%, respectively. The CYP2C19 wild type (*1/*1) accounted for 40.76% of the cases, while the mutant genotype accounted for 59.24% of the cases. A meta-analysis (31) predominantly involving a Western population found that the CYP2C19 wild-type genotype accounted for 71.5%, while the mutant genotype accounted for 28.5%. Compared to this, the proportion of the CYP2C19 mutant genotype was significantly higher in our population than in the Western population. For the ABCB1 gene, the TT type accounted for 19.48%, the CT type for 48.51%, and the CC type for 32.01%. The TRITON-TIMI 38 study (32) reported that 27% of ABCB1 genes were TT, 50% were CT, and 23% were CC. This indicates that the rate of the TT genotype was lower, and the rate of the CC genotype was higher in our population compared to the Western population. Regarding the PON1 Q192R gene, the wild-type (GG) allele was 32.50%, the heterozygous (GA) allele was 46.22%, and the mutant homozygous (AA) allele was 21.28%. Sibbing's study (33) reported 53.3% wild type, 38.9% heterozygous, and 7.8% mutant homozygous. Additionally, a previous study in Thailand reported a similar finding, with the CYP2C19*1 allele being the most prevalent at 70.14%, followed by the CYP2C19*2 and *3 alleles at frequencies of 25.36% and 4.50%, respectively. Conversely, the CYP2C19*3 allele was not detected in Caucasian, Hispanic, African, Italian, Macedonian, Tanzanian, or North Indian populations (34). In comparison, the rate of the mutant homozygous genotype was significantly higher in our study population than in the Western population. In summary, there are clear racial differences in gene polymorphisms. The high mutation rates at the five loci across the three genes studied, compared to the Western population, should be considered in assessing whether clopidogrel resistance could result. This is clinically important for further research on the effects of different gene polymorphisms on the efficacy of clopidogrel.

However, there is ongoing controversy regarding the effectiveness of antiplatelet therapy guided by genetic genotyping, particularly in patients undergoing percutaneous coronary intervention (PCI). Some studies suggest that selecting antiplatelet drugs based solely on the *CYP2C19* genotype does not offer additional clinical benefits. A multicenter prospective randomized clinical trial involving 5,302 PCI patients reported that gene-guided antiplatelet therapy did not significantly reduce the incidence of composite cardiovascular events compared to conventional therapy (35). A meta-analysis of 6,845 patients also failed to show the superiority of genotype- or phenotype-guided treatment over conventional methods (36). Furthermore, an observational study in Chinese patients with coronary artery disease indicated that *CYP2C19* genotype-based antiplatelet therapy did not improve clinical outcomes (37).

While *CYP2C19* phenotyping (NM, IM, PM) is widely recommended to guide clopidogrel therapy, our findings highlight its limitations as a solitary predictor. Nearly half of *CYP2C19* normal metabolizers in our cohort experienced MACE, with concurrent *ABCB1* or *PON1* variants explaining much of this residual risk. This underscores that *CYP2C19* phenotyping, though clinically implemented, cannot fully account for the polygenic determinants of clopidogrel response. Instead, our multigene model (*CYP2C19*, *ABCB1*, *PON1*) provides a more robust framework for risk stratification, aligning with the complex pharmacokinetic pathways governing clopidogrel efficacy. Thus, while *CYP2C19* remains a cornerstone of precision medicine, its integration with *ABCB1* and *PON1* genotyping is essential to optimize therapeutic outcomes.

However, in our previous study (shown in Supplementary Tables S3, S4), we found that when only *CYP2C19* metabolotypes were considered, 94 patients (47%) out of the 200 *CYP2C19* fast metabolizing population still experienced cardiovascular adverse events. Among these 94 patients, 67 (71.3%) had a *PON1* genotype of *GA*/*AA* and 61 (64.9%) had an *ABC1* genotype of *CT*/*TT*. In a subgroup analysis of 144 patients who returned for coronary angiography, it was found that 8 (13.33%) of the 60 fast metabolizing patients had in-stent restenosis when only the effect of a single *CYP2C19* gene was considered. Further examination of these eight patients revealed that 5 (62.5%) had the *PON1* genotype of *GA*/*AA* and 4 (50%) had the *ABC1* genotype of *CT*. This phenomenon attracted the attention of our group. Based on the existence of multiple genes with multiple loci involved in the clopidogrel metabolic pathway, we speculated that, in addition to *CYP2C19*, the *ABCB1* and *PON1* genes also play essential roles in its absorption and metabolism. This may explain the controversial results in previous studies that explored the efficacy of gene-guided antiplatelet therapy, which have mostly focused on analyzing individual *CYP2C19* genes or phenotypes. There may be other genes that influence the efficacy of antiplatelet drugs.

In the preliminary stages of this project as well, we analyzed the impact of *CYP2C19* metabolic phenotypes on the efficacy of clopidogrel. Multivariate analysis indicated that the *CYP2C19* metabolic phenotype did not significantly affect the occurrence of adverse cardiovascular events after clopidogrel treatment (*P* > 0.05) (shown in Supplementary Table S5). Consequently, we extended our investigation to include the combined effect of *CYP2C19* metabolic phenotypes with *ABCB1 C3435T* and *PON1 Q192R* polymorphisms on the efficacy of clopidogrel. We classified these multi-gene combinations into levels, and multivariate analysis revealed that Levels 2 [*P* = 0.003, HR = 1.983, 95% CI (1.267, 3.104)] and Levels 3 + 4 [*P* = 0.047, HR = 1.551, 95% CI (1.005, 2.393)] were independent risk factors for adverse cardiovascular events after clopidogrel treatment. Additionally, a previous study found no correlation between *ABCB1 C3435T* alone and cardiovascular events but noted an increased risk of cardiovascular events when *ABCB1 C3435T* was considered alongside CYP2C19*2.

These findings suggest that the combination of *CYP2C19*, *ABCB1*, and *PON1* genes is related to clopidogrel effectiveness, and that multi-gene testing may be more sensitive and specific in predicting clopidogrel efficacy than testing a single genotype alone. Although we did not directly compare the sensitivity, specificity, and cost-effectiveness of multi-gene testing with *CYP2C19* genotype testing in this study, our results indicate that patients with multi-gene testing levels of 2, 3, or 4 may have a higher risk of adverse cardiovascular events. Early medication adjustments based on multi-gene testing could potentially prevent these events and reduce the economic burden on patients. We will address these limitations and conduct further studies to provide a more comprehensive analysis of future research.

The novelty of this study lies in its comprehensive consideration of multiple genes related to the efficacy of clopidogrel. Besides *CYP2C19*, the *ABCB1* and *PON1* genes play crucial roles in the drug's absorption and metabolism. By integrating multiple genetic variants such as *CYP2C19* (*2, *3, *17), *ABCB1 C3435T*, and *PON1 Q192R*, we explored the effectiveness of antiplatelet therapy guided by multigene testing results in clinical practice. Our retrospective analysis and propensity score matching to control for confounding factors revealed that treatment plans consistent with multigene testing results significantly reduced the incidence of adverse cardiovascular events. This finding demonstrated the potential value of multigene testing in personalized antiplatelet therapy.

These findings indicate that clopidogrel efficacy is influenced by a complex mechanism regulated by multiple genes. Compared to single-gene testing, multigene testing can more comprehensively cover various aspects of clopidogrel's mechanism of action. It may provide a more refined molecular basis for developing individualized treatment strategies. Nevertheless, further prospective validation studies on a larger scale and in diverse populations are necessary to confirm whether multigene testing-guided antiplatelet treatment can offer significant statistical and clinical benefits. Additionally, other genetic and non-genetic factors might affect clopidogrel efficacy. Given that cardiovascular disease results from multifactorial and polygenic interactions, future research should further encompass genetic, phenotypic, environmental, and lifestyle factors to optimize individualized antiplatelet treatment strategies.

Despite its robust findings, this study has certain limitations. Firstly, while propensity score matching enhances the credibility of results from retrospective clinical trials, the single-center nature and limited sample size of this study may introduce a degree of bias and limit generalizability. Secondly, while propensity score matching balances known confounding factors, it cannot eliminate unknown confounders, nor can it prevent potential confounders that may be undetected in the study. Finally, the inability to record and compare new diseases, blood lipid levels, and liver and kidney function during follow-up periods means it is uncertain whether clinical data during follow-up influenced outcomes. More definitive conclusions await further confirmation by larger sample and multicenter real-world clinical studies.

In this study, we defined bleeding events using the criteria outlined in the published PLATO study (2009) (27), allowing for comparisons with previous research. However, there is a consensus on the standardized bleeding definitions for cardiovascular clinical trials as recommended by the Bleeding Academic Research Consortium (BARC) (38). Since the BARC criteria have standardized the definition of bleeding events in cardiovascular clinical studies, our study identified bleeding events through the use of hospital electronic medical records, telephone interviews, and rehospitalization data. This data collection method made it challenging to standardize the bleeding event definitions using the BARC criteria. For future studies, we recommend utilizing the BARC criteria to define outcome measures, ensuring that results are standardized and comparable across studies.

## Conclusion

This study indicates that antiplatelet treatment strategies based on multigene testing results (including variations in *CYP2C19*, *ABCB1*, and *PON1* genes) are more effective than those guided solely by the *CYP2C19* genetic profile. By employing propensity score matching, treatment plans conforming to multigene testing significantly reduced the incidence of adverse cardiovascular events, particularly in post-PCI patients. These findings underscore the importance of considering multiple genetic interactions when assessing clopidogrel responsiveness, providing a precise molecular basis for personalized treatment.

Further prospective research is required to validate the effectiveness and generalizability of multigene testing across diverse populations. Future studies should incorporate genetic, environmental, and lifestyle factors to develop a more comprehensive model for individualized medicine. Such an approach could enhance antiplatelet therapy efficacy and drive advancements in cardiovascular disease management, thereby improving patient quality of life and treatment outcomes.

In summary, the application of multigene testing in individualised cardiovascular disease management holds significant clinical relevance and is expected to become an integral part of future medical practices. Adopting this personalised approach can optimise antiplatelet therapy, mitigate risks, and ensure better patient care and outcomes.

## Data Availability

The original contributions presented in the study are included in the article/Supplementary Material, further inquiries can be directed to the corresponding authors.
