# Supplementary material for: Genetic and clinical determinants of MACE and haemorrhage in antiplatelet therapy: insights from pharmacogenomic analysis
Source: Front Cardiovasc Med. 2025 May 12;12:1572389. doi: 10.3389/fcvm.2025.1572389 (PMC12104257; doi:10.3389/fcvm.2025.1572389)
Supplement: Supplementary file 1 [file Datasheet1.pdf]

**Supplementary Table 1 Results of Multifactorial Cox Regression Analysis of MACE Before/After Adjustment by IPTW**

| Variable | Before IPTW | | | After IPTW | | |
|---|---|---|---|---|---|---|
| | HR | 95%CI | P | HR | 95%CI | P |
| Group (B) | | | | | | |
| A | 0.691 | 0.542-0.879 | 0.002* | 0.671 | 0.526-0.855 | 0.001* |
| Gender (Female) | | | | | | |
| Male | 0.711 | 0.498-1.012 | 0.058 | 0.701 | 0.487-1.008 | 0.055 |
| Age (<55years) | | | | | | |
| ≥55 and <65 | 1.102 | 0.809-1.502 | 0.534 | 1.093 | 0.796-1.501 | 0.582 |
| ≥65 and <75 | 1.860 | 1.338-2.585 | <0.001* | 1.940 | 1.356-2.775 | <0.001* |
| ≥75 | 1.362 | 0.877-2.115 | 0.167 | 1.393 | 0.905-2.144 | 0.131 |
| Nation (Han) | | | | | | |
| Uygur | 1.364 | 1.027-1.812 | 0.031* | 1.388 | 1.036-1.858 | 0.027* |
| Other | 1.695 | 1.193-2.409 | 0.003* | 1.692 | 1.142-2.507 | 0.008* |
| BMI (<24kg/m$^2$) | | | | | | |
| ≥24 | 1.311 | 0.985-1.745 | 0.062* | 1.277 | 0.935-1.742 | 0.123 |
| Smoke (No) | | | | | | |
| Yes | 1.504 | 1.117-2.026 | 0.007* | 1.546 | 1.127-2.121 | 0.006* |
| Diabetes (No) | | | | | | |
| Yes | 1.291 | 1.008-1.652 | 0.042* | 1.252 | 0.969-1.617 | 0.084 |
| Disease type (Chronic coronary syndrome) | | | | | | |
| Acute coronary | 1.025 | 0.802-1.309 | 0.843 | 1.005 | 0.787-1.282 | 0.966 |
| Surgical intervention (No) | | | | | | |
| PCI | 1.134 | 0.859-1.496 | 0.372 | 1.176 | 0.895-1.545 | 0.242 |
| CABG | 0.309 | 0.096-0.992 | 0.048* | 0.266 | 0.087-0.814 | 0.020* |

| Variable | Before IPTW | | | After IPTW | | |
|---|---|---|---|---|---|---|
| Monocyte percentage (<10.00%) | | | | | | |
| ≥10.00 | 1.497 | 1.053-2.128 | 0.024* | 1.452 | 1.037-2.033 | 0.029* |
| Platelet count(＜300.00×10^9/L) | | | | | | |
| ≥300.00 | 0.759 | 0.514-1.121 | 0.165 | 0.717 | 0.491-1.049 | 0.086 |
| Mean platelet volume (＜12.50fL) | | | | | | |
| ≥12.50 | 1.192 | 0.669-2.125 | 0.550 | 1.431 | 0.768-2.662 | 0.258 |
| Uric acid (<428.00μmol/L) | | | | | | |
| ≥428.00 | 1.614 | 1.121-2.324 | 0.009* | 1.646 | 1.148-2.360 | 0.006* |
| Low density lipoprotein (<1.80mmol/L) | | | | | | |
| ≥ 1.80 | 0.951 | 0.736-1.228 | 0.702 | 0.913 | 0.701-1.189 | 0.501 |
| Low left ventricular ejection fraction (≥50%) | | | | | | |
| <50 | 1.129 | 0.752-1.694 | 0.556 | 1.196 | 0.792-1.808 | 0.393 |
| Elevated risk of haemorrhage# (No) | | | | | | |
| Yes | 0.861 | 0.567-1.308 | 0.484 | 0.791 | 0.482-1.295 | 0.351 |

**Supplementary Table 2 Results of Multifactorial Cox Regression Analysis of Hemorrhage Before/After Adjustment by IPTW**

| Variable | Before IPTW | | | After IPTW | | |
|---|---|---|---|---|---|---|
| | HR | 95%CI | P | HR | 95%CI | P |
| Group (B) | | | | | | |
| A | 0.851 | 0.615-1.177 | 0.330 | 0.831 | 0.598-1.155 | 0.271 |
| Gender (Female) | | | | | | |
| Male | 0.935 | 0.599-1.459 | 0.768 | 0.884 | 0.552-1.414 | 0.608 |
| Age (<55years) | | | | | | |
| ≥55 and <65 | 1.137 | 0.750-1.723 | 0.544 | 1.104 | 0.735-1.658 | 0.631 |
| ≥65 and <75 | 1.317 | 0.832-2.085 | 0.239 | 1.227 | 0.749-2.010 | 0.415 |
| ≥75 | 1.121 | 0.624-2.011 | 0.703 | 1.077 | 0.581-1.998 | 0.813 |
| Nation (Han) | | | | | | |
| Uygur | 0.604 | 0.386-0.945 | 0.027* | 0.592 | 0.369-0.951 | 0.030* |
| Other | 1.226 | 0.771-1.951 | 0.387 | 1.333 | 0.821-2.164 | 0.244 |
| BMI (<24kg/m2) | | | | | | |
| ≥24 | 1.069 | 0.744-1.536 | 0.184 | 1.044 | 0.704-1.548 | 0.828 |
| Smoke (No) | | | | | | |
| Yes | 0.769 | 0.522-1.133 | 0.716 | 0.721 | 0.531-1.198 | 0.275 |
| Diabetes (No) | | | | | | |
| Yes | 0.751 | 0.529-1.068 | 0.111 | 1.044 | 0.498-1.043 | 0.082 |
| Disease type (Chronic coronary syndrome) | | | | | | |
| Acute coronary | 1.232 | 0.872-1.739 | 0.235 | 1.218 | 0.859-1.727 | 0.267 |
| Surgical intervention (No) | | | | | | |
| PCI | 1.633 | 1.087-2.452 | 0.018 | 1.723 | 1.147-2.588 | 0.008 |
| CABG | 0.309 | 0.041-2.301 | 0.251 | 0.385 | 0.051-2.887 | 0.353 |

| Variable | Before IPTW | | | After IPTW | | |
|---|---|---|---|---|---|---|
| | HR | 95%CI | P | HR | 95%CI | P |
| Monocyte percentage (<10.00%) | | | | | | |
| ≥10.00 | 0.966 | 0.559-1.669 | 0.903 | 0.927 | 0.512-1.678 | 0.802 |
| Platelet count(＜300.00×10^9/L) | | | | | | |
| ≥300.00 | 1.088 | 0.652-1.816 | 0.745 | 1.123 | 0.705-1.790 | 0.623 |
| Mean platelet volume (＜12.50fL) | | | | | | |
| ≥12.50 | 0.617 | 0.224-1.701 | 0.351 | 1.023 | 0.398-2.631 | 0.961 |
| Uric acid (<428.00μmol/L) | | | | | | |
| ≥428.00 | 1.575 | 0.961-2.581 | 0.071 | 1.638 | 1.121-2.324 | 0.067 |
| Low density lipoprotein (<1.80mmol/L) | | | | | | |
| ≥ 1.80 | 0.923 | 0.654-1.303 | 0.651 | 0.914 | 0.638-1.311 | 0.627 |
| Low left ventricular ejection fraction (≥50%) | | | | | | |
| <50 | 1.657 | 0.971-2.828 | 0.064 | 1.636 | 0.964-2.782 | 0.075 |
| Elevated risk of haemorrhage# (No) | | | | | | |
| Yes | 0.982 | 0.570-1.691 | 0.947 | 0.901 | 0.519-1.561 | 0.710 |

**Supplementary Table 3 Univariate analysis results of subgroup genetic data (single gene) n(%)**

| Factors | Totle No | Treatment for in-stent restenosis after therapy | Log-rank test |
|---|---|---|---|
| | | | *P* |
| CYP2C19 genotype | | | 0.080 |
| Ultra-Rapid Metabolizer | 11 | 4(36.36) | |
| Extensive Metabolizer | 60 | 8(13.33) | |
| Intermediate Metabolizer | 61 | 13(21.31) | |
| Poor Metabolizer | 12 | 3(25.00) | |
| ABCB1genotype | | | 0.754 |
| CC | 45 | 9(20.00) | |
| CT | 69 | 15(21.74) | |
| TT | 30 | 4(13.33) | |
| ABCB1genotype combined | | | 0.686 |
| CC | 45 | 9(20.00) | |
| CT+TT | 99 | 19(19.19) | |
| PON1genotype | | | 0.613 |
| GG | 50 | 9(18.00) | |
| GA | 64 | 13(20.31) | |
| AA | 30 | 6(20.00) | |
| PON1genotypecombined | | | 0.742 |
| GG | 50 | 9(18.00) | |
| GA+AA | 94 | 19(20.21) | |

**Supplementary Table 4 Univariate analysis results of subgroup genetic data (gene combination) n(%)**

| Factors | Totle No | Treatment for in-stent restenosis after therapy | Log-rank test *P* |
|---|---|---|---|
| **CYP2C19 and ABCB1 Combination** | | | 0.121 |
| CYP2C19 (Ultra-Rapid Metabolizer + Extensive Metabolizer) + ABCB1 CC | 22 | 6(27.27) | |
| CYP2C19 (Ultra-Rapid Metabolizer + Extensive Metabolizer) + ABCB1 CT/TT or CYP2C19 (Intermediate Metabolizer + Poor Metabolizer) + ABCB1 CC | 72 | 9(12.50) | |
| CYP2C19 (Intermediate Metabolizer + Poor Metabolizer) + ABCB1 CT/TT | 50 | 13(26.00) | |
| **CYP2C19 and PON1 Combination** | | | 0.378 |
| CYP2C19 (Ultra-Rapid Metabolizer + Extensive Metabolizer) + PON1 GG | 21 | 4(19.05) | |
| CYP2C19 (Ultra-Rapid Metabolizer + Extensive Metabolizer) + PON1 GA/AA or CYP2C19 (Intermediate Metabolizer + Poor Metabolizer) + PON1 GG | 79 | 13(16.46) | |
| CYP2C19 (Intermediate Metabolizer + Poor Metabolizer) + PON1 GA/AA | 44 | 11(25.00) | |
| **ABCB1 and PON1 Combination** | | | 0.867 |
| ABCB1 CC + PON1 GG | 19 | 3(15.79) | |
| ABCB1 CC + PON1 GA/AA or | 57 | 12(21.05) | |
| ABCB1 CT/TT + PON1 GG | 68 | 13(19.12) | |
| ABCB1 CT/TT + PON1 GA/AA | | | 0.136 |
| **CYP2C19, ABCB1, and PON1 Combination** | 26 | 7(26.92) | |
| Level -1 (Bleeding) + Level 0 (Normal) | 54 | 7(12.96) | |
| Level 1 (Low Risk) | 26 | 5(19.23) | |
| Level 2 (Moderate Risk) | 38 | 9(23.68) | |

**Supplementary Table 5 Results of Preliminary study on the impact of CYP2C19 metabolic phenotypes on the efficacy of clopidogrel**

| Factors | β | Wald | HR | 95%CI | *P* |
|---|---|---|---|---|---|
| Age (year) | | | | | |
| 55-65 | 0.165 | 0.798 | 1.180 | 0.821~1.696 | 0.372 |
| 65-75 | 0.629 | 10.580 | 1.875 | 1.284~2.739 | 0.001 |
| ≥75 | 0.283 | 1.116 | 1.327 | 0.785~2.243 | 0.291 |
| Gender (reference Male) | | | | | |
| Female | -0.130 | 0.335 | 0.878 | 0.566~1.363 | 0.563 |
| Ethnics (reference Han) | | | | | |
| Uygur | 0.462 | 7.553 | 1.587 | 1.142~2.206 | 0.006 |
| Other | 0.468 | 4.866 | 1.597 | 1.054~2.421 | 0.027 |
| BMI ≥24(kg/m$^2$) | 0.342 | 4.114 | 1.408 | 1.012~1.960 | 0.043 |
| Smoking (reference No) | 0.313 | 3.160 | 1.367 | 0.968~1.930 | 0.075 |
| Diabetes (reference No) | 0.138 | 0.919 | 1.148 | 0.866~1.523 | 0.338 |
| Percentage of neutrophils ≥75 (%) | 0.370 | 1.672 | 1.448 | 0.826~2.539 | 0.196 |
| Monocyte percentage ≥10 (%) | 0.546 | 6.849 | 1.727 | 1.147~2.600 | 0.009 |
| Lymphocyte count ≥1.10 (10^9/L) | -0.215 | 0.473 | 0.806 | 0.436~1.490 | 0.492 |
| Low red blood cell count <4.30 (10^12/L) | 0.230 | 1.421 | 1.259 | 0.862~1.839 | 0.233 |
| Low haemoglobin <120 (g/L) | 0.366 | 2.093 | 1.441 | 0.878~2.365 | 0.148 |
| Uric acid ≥428 (μmol/L) | 0.380 | 3.056 | 1.462 | 0.955~2.240 | 0.080 |
| Total protein ≥65 (g/L) | -0.195 | 1.740 | 0.823 | 0.616~1.099 | 0.187 |
| Alanine aminotransferase ≥50 (U/L) | -0.419 | 2.588 | 0.658 | 0.395~1.096 | 0.108 |
| Low-density lipoprotein ≥1.80 (mmol/L) | 0.039 | 0.064 | 1.040 | 0.768~1.407 | 0.800 |
| Low left ventricular shortening fraction <25 (%) | 1.080 | 2.254 | 2.945 | 0.719~12.059 | 0.133 |
| Low left ventricular ejection fraction <50 (%) | -0.715 | 1.030 | 0.489 | 0.123~1.946 | 0.310 |
| CYP2C19 metabolic phenotype | | | | | |
| Ultrafast metabolism vs (fast metabolism) | -0.051 | 0.029 | 0.951 | 0.531~1.701 | 0.865 |
| Intermediate metabolism vs (fast metabolism) | 0.281 | 3.592 | 1.324 | 0.990~1.771 | 0.058 |
| (slow metabolism) vs (fast metabolism) | 0.269 | 0.907 | 1.309 | 0.752~2.277 | 0.341 |
